# Response of soil microbiome structure and its network profiles to four soil amendments in monocropping strawberry greenhouse

**DOI:** 10.1371/journal.pone.0245180

**Published:** 2021-09-29

**Authors:** Senlin Liu, Muzammil Hassan Khan, Zhongyuan Yuan, Sarfraz Hussain, Hui Cao, Yabo Liu

**Affiliations:** 1 College of Life Sciences/Key Laboratory of Agricultural Environmental Microbiology, Ministry of Agriculture and Rural Affair, Nanjing Agricultural University, Nanjing, P. R. China; 2 Zhenjiang Institute of Agricultural Sciences, Jurong, China; Dong-A University, REPUBLIC OF KOREA

## Abstract

With the constant surge of strawberry cultivation and human demand, widespread concern has been expressed about the severe soil and plant health problems caused by continuous strawberry cropping, particularly monocropping in greenhouses. Effective microorganisms (EM) and Bacillus subtilis (BS) have been extensively commercialized as biological control agents (BCAs) to promote plant growth and yield enhancement. However, their effects on soil microbes are obscure. To regulate the microbial community in continuous cropping strawberry soils, we developed four soil amendments based on these two BCAs by adding low and high contents of compost. The amplicon sequencing of bacterial and fungal ribosomal markers was applied to study the response of the soil microbiome structure. We noticed a sharp increase in bacterial diversity after adding EM-treated high compost and BS-treated low compost, while there was no significant change in fungal diversity among treatments. Through taxonomic classification and FUNGuild analysis, we found that the application of soil amendments resulted in a significant decline in the relative abundance of fungal plant pathogens (*Rhizopus*, *Penicillium* and *Fusarium*) in the soils; accordingly, the metabolic functions of a range of detrimental fungi were inhibited. Correlation analysis indicated that soil microbial community was indirectly driven by soil physicochemical properties. Co-occurrence networks revealed that soil amendments contributed to the connectivity of bacterial network, and EM-treated with high compost was the most complex and balanced. Collectively, EM-treated high compost and BS-treated low compost can well regulate the microbial community structure and thus maintain soil health.

## 1. Introduction

Strawberries (*Fragaria ananassa Duchesne*) are world-renowned high-value soft fruits. China’s strawberry cultivation area accounts for 40% of the world’s total in 2016. With a total production of more than $10 billion, the industry is one of the major contributors to the national economy [1, 2]. Soil microbes play essential roles in maintaining soil health and ecosystem function [3]. Long-term monocropping on the same site may cause serious problems [4], which could result in changes in soil microbial community composition, particularly in pathogenic microbial accumulation and a reduction in the abundance of beneficial microbes [5, 6]. For instance, over the past decade, there has been a significant decline in the richness and diversity of bacterial and fungal communities and a significant increase in the communities of *Fusarium* in the continuous cropping strawberry fields [7]. According to recent research, the growing problem of monocropping in strawberry production is prevalent in all regions [3, 8].

Anaerobic soil disinfection (ASD), is commonly applied as a soil chemical fungicide in the plant cultivation [9]. It has been proven to block the spread of soil-borne plant pathogens in the field settings, thereby improving crop yields [10]. Until 2018, a field trial conducted by [11] showed that ASD induces changes in soil microbiome structure and strawberry disease-causing pathogens, and enhances commercial strawberry production. However, the fact that the pathogen can survive in the soil for years makes soil fungicide only partially effective [12]. Crop rotation is also known to be an option to mitigate soil pathogens. The increase in yield of corn-soybean rotation is usually attributed to microbial community in the soil, especially when it comes to disease control and nutrient availability [13, 14]. However, these traditional methods have many drawbacks, for instance environmental pollution and high costs. Consequently, we have further to address this problem with more economical and safety-friendly soil amendments.

In general, beneficial soil microbes can compete with pathogens [14, 15]. Furthermore, these microbes help manage nutrients by making nutrients available in plants through decomposition, solubilization, iron carrier production, or symbiosis [16]. A series of studies have shown that organic amendments usually have the most significant effects on microbial community in agricultural soils, such as compost or manure [17, 18]. Therefore, the use of soil amendments based on biological control agents (BCAs) and compost is considered to be a sustainable strategy [19, 20].

According to the EM Research Organization (www.emrojapan.com/how/), Effective Microorganism (EM) agent has been developed in Japan since the 1980s, and it has been confirmed to be composed of lactic acid bacteria, yeasts, nitrogen-fixing bacteria, and photosynthetic bacteria [21]. It has been reported that EM agent could increase the diversity of soil microbes and control soil diseases, thus contributing to crop growth [22, 23]. Laura et al. revealed that combining EM agents and compost could enhance the resistance of soil food webs to drought stress even while improving nitrogen mineralization from compost manure [24]. Notably, the successful performance of EM agent depends on appropriate formulation techniques and ingredients (nutrients, adhesives) for improving its durability and reliability [25]. As another BCA in soil amendment used, *Bacillus subtilis*, scientists have found that it has a good inhibition effect on various plant pathogens [26], including *Verticillium sp*, *Fusarium oxysporum* and *Penicillium digitatum* [27, 28]. Besides, scientists studied the impact of incorporating *Bacillus subtilis* on the composition of bacterial and fungal communities in the cucumber and rice rhizosphere. They found that it could be used as a plant protection agent compatible with the soil environment, depending on the soil type [8, 29].

Hitherto, numerous studies have focused on the effects of fertilization and soil management measures, including tillage [30], rotation [31], straw [32]. Besides, relevant studies on the addition of soil amendments in the strawberry field mainly gave priority to its pathogenic fungi. It is well known that soil microbes are not isolated within microbial communities and those complex interactions exist between them [33]. Co-occurrence network analysis, the popular new ecological model in recent years, has greatly improved human understanding of soil microbial interactions and metabolic potential, in addition to facilitating the understanding of the niche space of community members [34, 35]. In contrast, there are few studies on the function and co-occurrence network of strawberry soil microbes [36, 37].

In this study, we applied soil amendments technqiue based on two popular commercials BCAs by adjusting composting ratios. A field experiment was carried out in a long-term monocropping strawberry greenhouse in southern China, and we applied 16S rRNA amplicon sequencing [38] for further study. We hypothesize that, with soil amendments processing, co-occurrence patterns between strawberry soil microbiomes could be ideally improved (correlated connectivity and density of microbial networks are increased). Besides, we assume that the soil ecological function has predictable heterogeneity. The objectives of our study are: (1) to evaluate the effect of soil amendments on the structure of soil microbial communities in monocropping field; and (2) to put forward a theoretical and practical basis for the sustainable production of strawberries and other plants from the perspective of microbial ecology.

## 2. Materials and methods

### 2.1. Soil amendments preparation

The soil amendments compared were organic compost with two biological control agents (BCAs): Effective Microorganisms (EM) and *Bacillus subtilis* (BS). Rice bran and soybean meal were blended clinched alongside a ratio of 1:2 (dry weight) to serve compost for the processing of soil amendments [39]. The specific status and appearance of the two commercial BCAs are shown in S1 Fig. The EM (~1×10^9^CFU/mL) and BS (~1×10^11^CFU/mL) are produced by Jiangsu Warner Biotechnology Co., Ltd. The main EM components were *lactobacillus plantarum*, *Lactobacillus acidophilus*, *Lactobacillus pentose*, yeast (e.g., *Saccharomyces spp*.), *Bacillus pumilus*, nitrifying bacteria and metabolites [40]. The main component of BS is *Bacillus subtilis*. The original EM agent is of pH 4.0 compared to 7.0 for BS. EM was activated by using 200 mL mother culture EM•1® and mixed with 50 mL unsulfured molasses in 200-fold non-chlorinated under anaerobic conditions [41]. According to the literature, the minimum rate of EM use is 1 L/m2 [42]. After a week, activated EM can be used in agricultural fields when the pH is 3.3–3.4 [41]. For BS agent, it was diluted in 200-fold ratio with non-chlorinated water, and the status of the agent was measured by the OD600 (value 0.6) of the liquid. To obtain the optimized soil amendment, we added low (12.5kg) and high (25kg) content of compost to each BCA. Following preparation, the soil amendments were stored and then utilized within greenhouse experiments.

### 2.2. Experimental design and sampling

Our greenhouse experiment and design were carried out in Baitu Town, Zhenjiang City, Jiangsu Province, China (31°57’N, 120°09’E). The region has a Northern Subtropical climate, with average annual precipitation of 1022 mm and mean yearly temperature of 17.1°C [1].

The plots in the greenhouse are arranged in a randomized block design with three replications per treatment, and no application of soil amendments as the control. The greenhouse area was 10 m wide and 60 m long, which contained five experimental plots for five treatments, and each plot in the greenhouse is 0.5 m wide and 12 m long. As stated by those extent for unit zone utilized within greenhouse, the specific treatments were control, EM1 (1L/m^2^ + low content compost), EM2 (1L/m^2^ + high content compost), BS1 (BS 1L /m^2^ + low content compost) and BS2 (BS 1L /m^2^ + high content compost), respectively. Strawberries are planted in double rows with 20cm intervals, and the soil type is loam according to the Soil Classification Retrieval System of China.

Before this experiment, this greenhouse has been used to plant strawberries for more than five years; hence we chose it as the subject for study with potential soil health risks. As a traditional method of alleviating monocropping pathogens, the greenhouse was closed in July 2018 to make use of sunlight, and weeds were removed in August. Strawberries were transplanted in September, while soil amendments were introduced to the soil layer and covered with an agricultural plastic film according to the treatment process detailed in 2.1. Water was conveyed through drip irrigation and maintained under the same agricultural management model (S1 Fig). It was guaranteed the strawberries cultivated in greenhouse belonged to the same variety (*Benihoppe*).

In each treatment plot, we randomly collected three soil cores (5 cm diameter ×20 cm length) from the 0–20 cm layer as three replicates. In total, 15 soil samples were collected from five plots (Control, EM1, EM2, BS1, and BS2) in December 2018, when the strawberries were in the fruiting stage. Afterwards, the soils in aseptic plastic bags were transferred to the laboratory after removing the plant roots and stones. Each sample was divided into two portions: the soil stored at 4°C for physicochemical analysis and the other at -80°C for DNA extraction.

### 2.3. Analysis of soil physicochemical properties

Soil pH (soil:water = 1:5, w/v) was determined using a pH meter with a glass electrode (FE20-Five Easy Plus™, Switzerland) [43]. Total organic C(TOC) was determined according to the vitriol acid-potassium dichromate oxidation method [44]. Total nitrogen (TN) was measured based on direct combustion using an elemental analyzer [45]. C/N ratios were measured by the ratio of TOC to TN. Inorganic N (NH_4_^+^-N and NO_3_^−^-N) of soil was drawn with 2 mol/L KCl (soil: KCl = 1:10, w/v) by shaking (1h, 200 rpm) and filtering through polysulfone membrane before colorimetric determination requiring a continuous-flow analyzer [46]. Available K (AK) and total K (TK) of the soil samples were identified using flame photometry method [47], and available P (AP)was tested by the molybdenum blue method. Soil physicochemical properties are presented in S1 Table.

### 2.4. DNA extraction and 16S rRNA and ITS amplicon sequencing

In order to ensure the validity of the experiment, the soil was kept in the refrigerator for only one week before the soil DNA was extracted. The microbial DNA of fifteen soil samples was extracted from 1.0 g of each sample by the E.Z.N.A.® Soil DNA Kit (Omega Bio-tek, Norcross, GA, U.S.) conforming to the manufacturer’s instructions. The V3-V4 region of the 16S rRNA gene and the ITS1 region of the fungal ITS gene were selected as specific fragments for detection of bacteria and fungi using primers 338F/806R [48] and ITS1F/ITS2 [49], respectively. PCR reactions were performed in triplicate 30 μl mixtures containing 10 ng of template DNA, Phusion® High-Fidelity PCR Master Mix (New England Biolabs) 15μl, 2μmol/L Primer 3μl. The PCR reactions for the 16S V3-V4 rRNA gene were conducted following the process: initial denaturation under 95°C for 3 min, 30 cycles consisting of denaturation for 30s at 95°C, annealing at 56°Cfor 30 s, followed by 72°C for 45 s, and a final extension for 5 min at 72°C; as for the ITS gene, the following procedure was followed: an initial denaturation step at 95°C for 3 min, followed by 35 cycles at 94°C for 30 s, 55°C for 30 s and 72°C for 45 s, and finally an extension of 10 min at 72°C[50]. The resulted PCR products were extracted from a 2% agarose gel and further purified with GeneJET TM Gel Extraction Kit (Thermo Scientific) as stated by the protocol of manufacturer. The library quality was assessed by the Qubit@ 2.0 Fluorometer (Thermo Scientific).

Single-end of 16S rRNA gene and ITS1 gene sequenced on an Ion S5TM XL platform (Wang et al., 2018) by Novogene Genomics Institute (Beijing, China). These raw reads are banked in the NCBI Sequence Read Archive (SRA) database under the accession PRJNA633325.

### 2.5. Bioinformatic processing and analysis

Microbiome data obtained from Ion S5TM XL sequencing was performed using QIIME (V2.0, http://qiime.org/) [51]. Single-end sequences were demultiplexed using the demux plugin. Then, quality control was performed on each sample using the Dada2 plugin. Then, sequences were quality controlled (> 25 score and the length of 200 bp), and according to the corresponding barcode assigned to different samples. Alpha‐diversity metrics (ACE, Chao1, Shannon and Simpson index), beta diversity metrics (Bray‐Curtis dissimilarity) were estimated using q2‐diversity after samples were rarefied to 23000 sequences per sample. Sequences at a 97% sequence identity were clustered into operational taxonomic units (OTUs) by q2-vsearch [52], then the bacterial OTUs of the representative sequences were performed by the Silva (Version 132) database (https://www.arb-silva.de/) [53]. The Heatmap, Barchart and correlation analysis (RDA, CCA) were displayed with R-Studio (Version 3.6). We defined specific OTUs as "abundant" when their average relative abundances were above 0.05% across all samples following [54]. The Mantel test focused on those soil physicochemical properties that significantly correlated with abundant OTUs by the Bray-Curtis dissimilarity algorithm. The differences between treatments were analysed by one-way ANOVA (P < 0.05) using the SPSS 25.0 software. We have uploaded the analysis code and files for the bacterial and fungal sequencing data to GitHub (account and repository: 18351000770LSL /Response-of-Soil-Microbiome-Structure-and-Its-Network-Profiles-to-Four-Soil-Amendments-in-Monocroppi).

Functional Annotation of Prokaryotic Taxa (FAPROTAX version 1.1) [55] was employed to annotate the functional annotation of bacterial communities in the normalized OTU table. FAPROTAX (http://mem.rcees.ac.cn:8080/root) is a manually constructed database that maps prokaryote to possible ecological functions (nitrification, denitrification or fermentation) or metabolic. For instance, if all cultured strains of the bacteria have been identified as nitrification types, FAPROTAX assumes that all uncultured genera are the same functional group [56]. Correspondingly, FUNGuild is an ITS-based functional prediction software launched in 2016 and is currently based on a classification prediction called ’guild’, which is based on data integrated from published literature [57]. There are 12 categories of pathogenic bacteria, animal pathogens and wood decay fungi.

### 2.6. Co-occurrence network analyses

In order to illustrate the co-occurrence interaction between bacteria in strawberry greenhouses, network analysis was performed on the abundance of the top 80 genera between treatments. We adopted Spearman’s correlation to obtain the strong correlation (r> | 0.8 |) and significant correlation (P <0.05) between taxa. Next, we used Cytoscape version 3.8.0 [58, 59] to visualize the network structure. The size of each node stands for relative abundance of the genus. The color of each node was distinguished depending on the level of phylum. The correlation was shown as an edge (positive correlation = grey; Negative correlation = red); At the same time, Gephi (v.0.9.2) and Network Analyzer were utilized to calculate the obtained network topology parameters (number of nodes and links, network density, shortest paths, network diameter, average neighbors, and clustering coefficient) to represent the co-occurrence relationship between genera [60].

## 3. Results

### 3.1. Richness and diversity of bacterial and fungal communities

**Fig 1:**
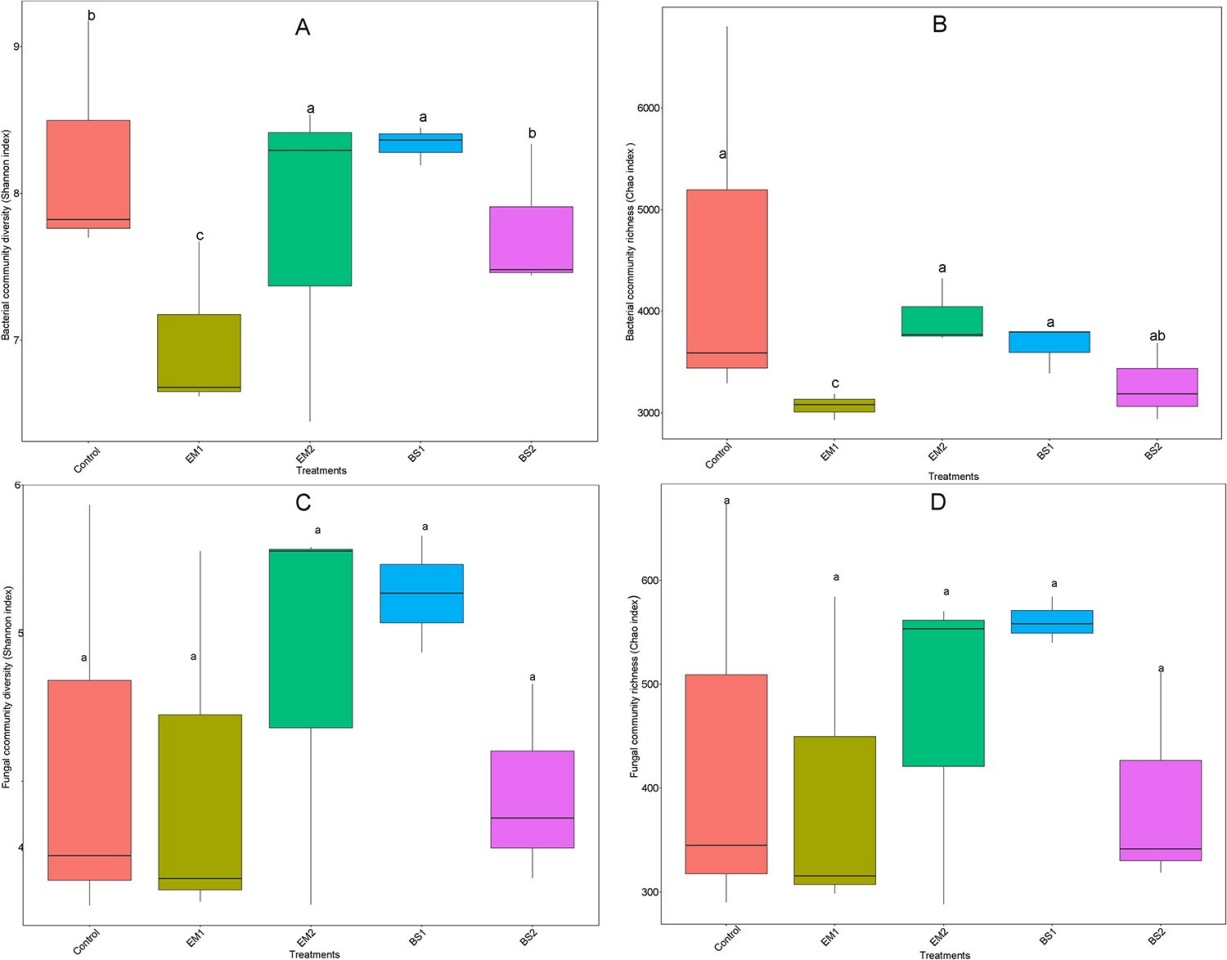
Comparative analysis of the alpha diversity index in different treated soils. (a) Shannon of bacterial 16S rRNA gene, (b) Chao of bacterial 16S rRNA gene, (c) Shannon of fungal ITS gene, (d) Chao of fungal ITS gene, were calculated by five treatments. Statistically significant differences were determined by one-way ANOVA (P < 0.05).

From 15 soil samples, we obtained a total of 1,537,746 high-quality V3-V4 sequences of 16S rRNA and 1,202,670 high-quality ITS1 sequences, average read length of bacteria and fungi were 437 and 282 bp, respectively. The sequences were grouped into 3888 bacterial operational taxonomic units (OTUs) and 1234 fungal OTUs at 97% sequence similarity cutoff (S1 File). For the α-diversity, indexes including observed OTUs, Chao1, ACE, Shannon, and Simpson of bacterial and fungal communities were observed (S2 Table). All coverage of soil bacteria and fungi was more than 97.9%, indicating the current sequencing depth in this study was accurate and reliable. The Shannon index (Fig 1A) showed that the bacterial diversity of the EM2 and BS1 treatments was significantly higher than that of the control (P < 0.01); the Chao index (Fig 1B) showed that the bacterial richness of EM1 was significantly lower than that in control (P < 0.05). Nevertheless, we observed no significant differences in the diversity and community richness of the soil fungi (Fig 1C and 1D).

**Fig 2:**
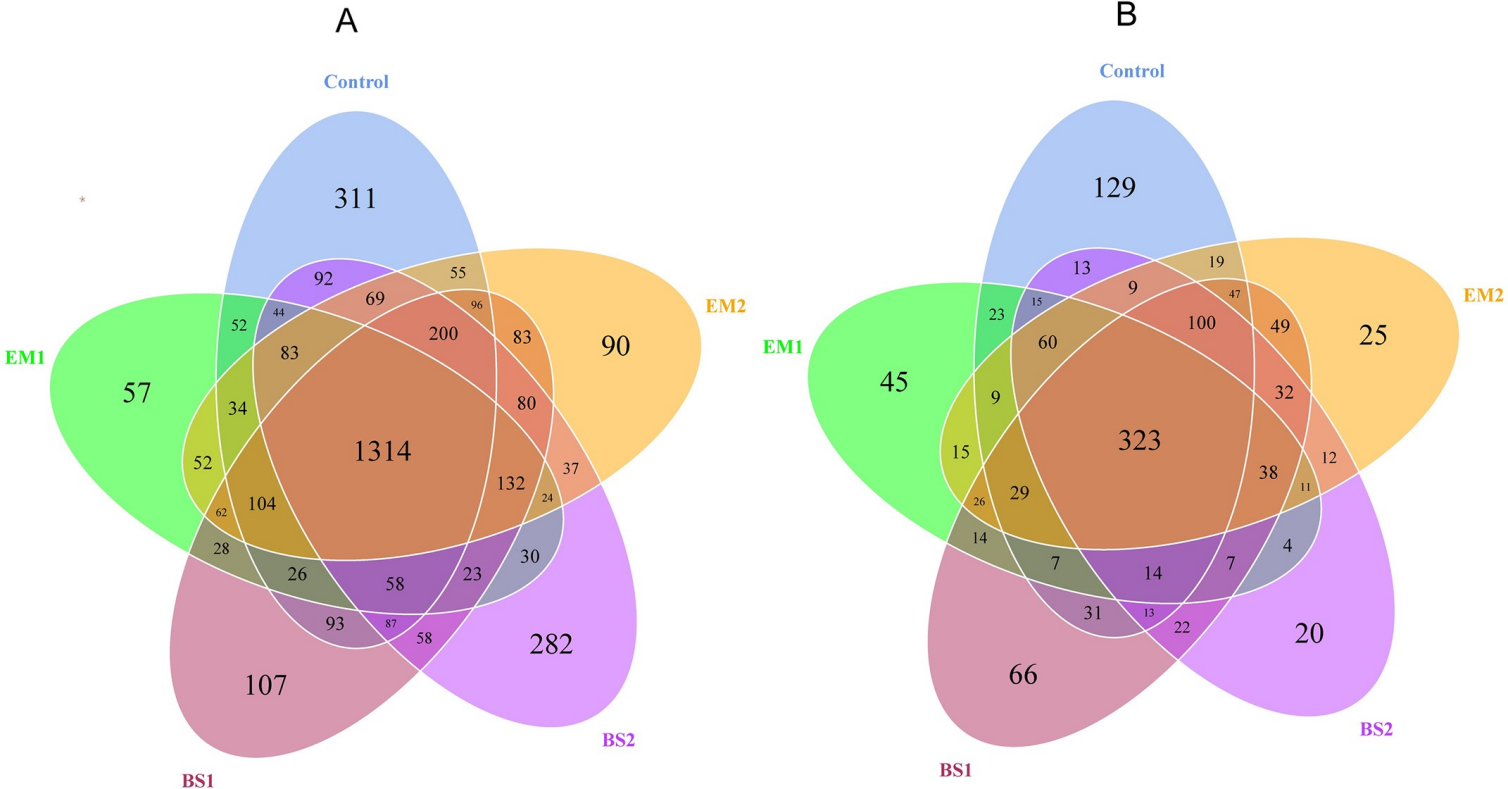
Shared OTUs analysis of the different libraries. (A) Venn diagram of bacterial OTUs between five treatments; (B) Venn diagram of fungal OTUs between five treatments. The numbers in one circle denote unique OTUs, and numbers in two or more intersecting circles denote shared OTUs.

The Venn diagram shows that the distribution of OTUs in the microbial community varied among the different treatments (Fig 2). A total of 1314 OTUs were shared among the five soil treatments, accounting for 33.80% of the total. In addition, 311, 57, 90, 107 and 282 OTUs were unique in the control, EM1, EM2, BS1 and BS2 treatments, respectively (Fig 2A). Interestingly, in the same BCA, the number of unique bacterial OTUs in the higher composts increased significantly. Soil fungi shared 323 OTUs among the five treatments, accounting for 26.16% of the total OTUs. In the order of the above soil treatments, there were 129, 45, 25, 66 and 20 unique OTUs for soil fungus, respectively. (Fig 2B). We found the significant reduction in the number of unique OTUs in compost with the same agent.

### 3.2. The taxonomic classification of core microbiome

**Fig 3:**
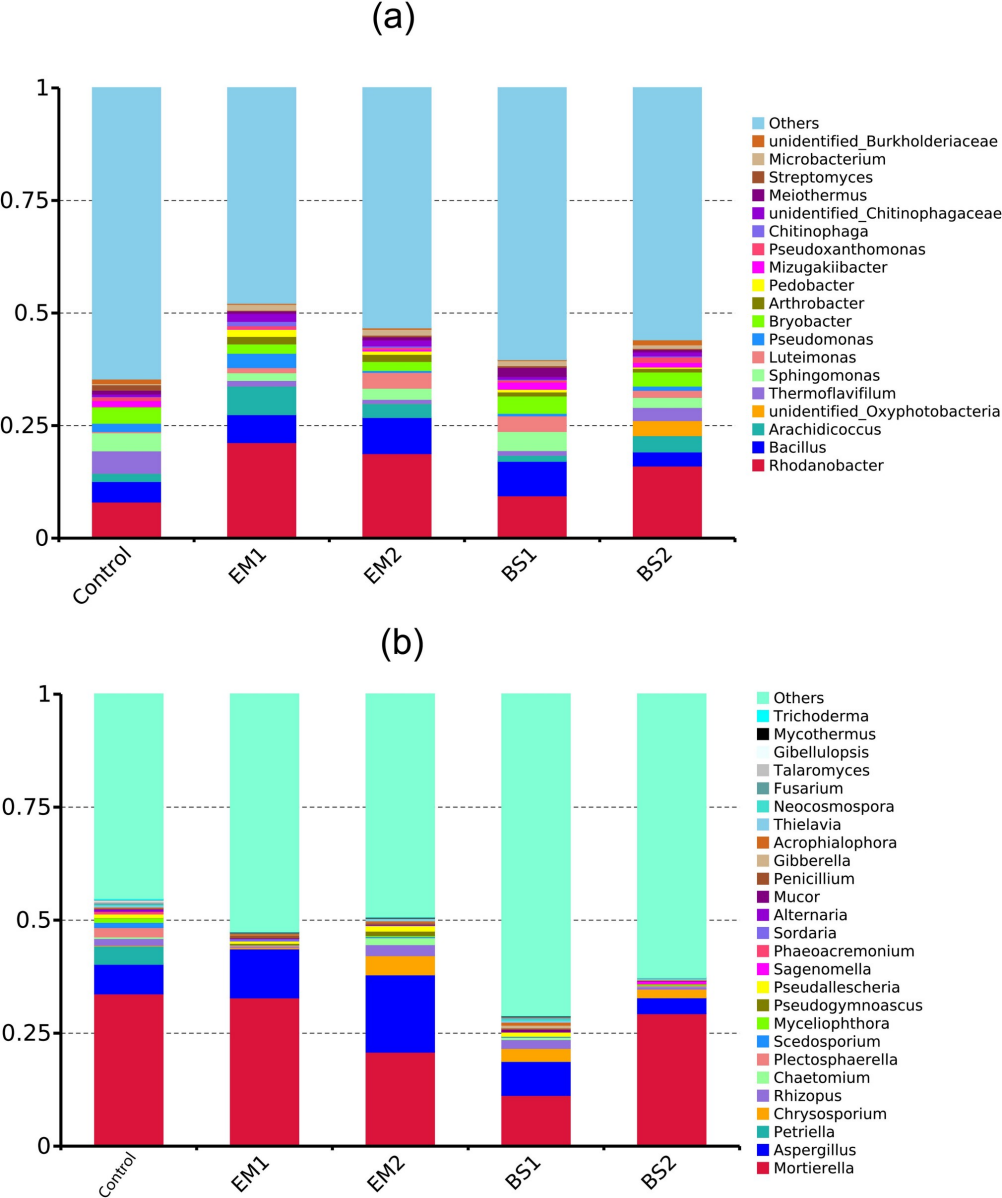
The taxonomic classification of core microbiome. Changes in the relative abundances of bacterial (a) and fungal (b) dominant genera under different treatments of strawberry soil, proportional distribution of taxa with abundance >1%.

According to the taxonomic identification, 42 phyla, 50 classes, 114 orders, 223 families, and 570 genera in bacterial community, while fungal OTUs could be classified into 10 phyla, 29 orders, 62 orders, 101 families and 144 genera. The composition of dominant (>1% relative abundance) bacterial and fungal communities in different soil samples was compared at the genus level, and 19 bacterial and 26 fungal genera were identified (Fig 3). For the bacterial communities, the most abundant genera were *Rhodanobacter* (8% - 21%), *Bacillus* (3% - 8%), and *Arachidicoccus* (1% - 6%). All four soil amendment treatments increased the total relative abundance of 19 genera, with greater changes of 47.46% for EM1 and 32.03% for EM2 relative than the control treatment (35.40%) (S3 Table). Further comparisons revealed that treatment with biocides resulted in significant changes in the relative abundance of five bacterial genera in strawberry soil. Concretely, the application of EM1, EM2 and BS2 increased the relative abundance of Rhodanobacter (p__Proteobacteria) and Arachidicoccu compared to the control treatment; specifically, EM1 increased the proportion of these two genera by 161% and 248%, respectively. Conversely, EM1, EM2 and BS2 significantly reduced the relative abundance of Sphingomona (p__Proteobacteria) from 4.02% in control to 1.71%, 2.47% and 2.23%, respectively. In the *Bacillus subtilis* treatments (BS1 and BS2), we did find a significant increase in the relative abundance of *Bacillus* (Firmicutes). Besides, all four soil amendment treatments significantly reduced the relative abundance of Thermoflavifilum (Bacteroidetes), with the most notable decrease in EM2 (S3 Table).

Considering that fungi are key microbes for soil-borne diseases, it is necessary to focus our attention on them. After a previous classification in the literature, we identified nine genera of plant pathogenic fungi in the monocropping soil (S4 Table), of which the first seven genera (including *Aspergillus*, *Rhizopus* and *Penicillium*, etc.) are dominant genera (Fig 3B) and the remaining two genera are rare taxa. The average total abundance of these nine pathogenic genera among treatments were 9.94% (Control), 0.98% (EM1), 8.27% (EM2), 3.60% (BS1) and 0.93% (BS2), respectively. The total abundance of these pathogenic fungi decreased with the application of the soil amendments, with EM1, BS2 decreasing the most. Further ANOVA revealed that seven fungal genera (*Aspergillus*, *Rhizoctonia*, *Penicillium*, *Fusarium*, *Alternaria*, *Mucor* and *Botrytis*) were found to be remarkably different (S4 Table); in particular, the relative abundance of *Rhizopus*, *Penicillium* and *Fusarium* all decreased significantly (p < 0.05) after the implementation of soil amendments.

### 3.3. Correlations of abundant microbial taxa with environmental variables

**Fig 4:**
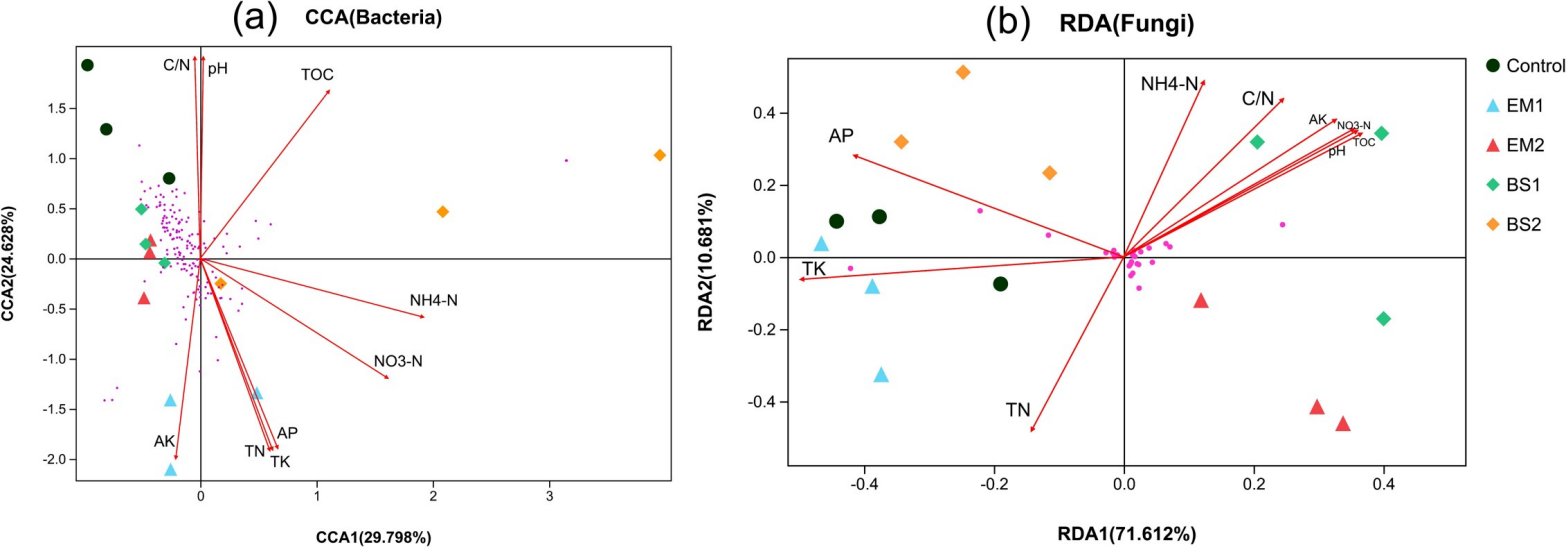
Correlations of abundant microbial taxa with environmental variables. RDA and CCA demonstrating the relationships between soil environmental factors and soil microbial communities(bacterial (a), fungal (b)) after application of soil amendments. The soil microbial communities selected the abundant OTUs represented by more than 0.5% relative abundance. The length of each arrow indicates the contribution of the corresponding parameters to the structural variation. The treatments are indicated in different colors respectively. Soil factors indicated in blue text include total carbon (TOC), total nitrogen (TN), total phosphorus(TP), NH_4_^+^-N, NO_3_^—^N, pH, Total potassium(TK), Available potassium(AK), Available phosphorus(AP).

The correlation analysis consisted of the soil physicochemical properties (for instance, TOC, pH, NH4^+^ and TN) and abundant microbial taxa (bacteria and fungi) at the OTU level (Fig 4). In the whole data set, the abundant taxa contained 159 bacterial OTUs and 28 fungal OTUs (purple dots).

The first two axes of the CCA explained 54.43% of the total variance in the abundant bacterial 16S rRNA genotypes composition (Fig 4A). The variation in bacterial composition was significantly explained by TN, AP, AK and C/N ratios. Following Fig 4A, the samples from EM2 and BS1 were found to be close to more bacterial OTUs, which means that these treatments occupied richer bacterial communities, consistent with previous findings. In comparison, rarer bacterial communities were present in the EM1 samples, and they all correlated positively with TN, AP and AK. For the fungal communities (Fig 4B), RDA explained 82.293% of the total variation within the abundant OTUs. Within these communities, the different treatments were clearly distinguished from each other. We also found that BS2 samples were positively correlated with AP; both control and EM1 were positively correlated with TK. Interestingly, the fungi in BS1 were strongly positively correlated with most of the other soil physicochemical properties. However, the significance of these correlations needs to be further explored.

Mantel test (S5 Table) revealed significant (P = 0.008, 0.003 and 0.002, 0.037, P-values based on 999 permutations) relationships between abundant bacterial community and TN, AP, AK and C/N ratios, respectively. On the other hand, only two soil physicochemical characteristics (AP and TK) have significant relationships (P = 0.001 and 0.002) with abundant fungal communities among treatments.

### 3.4. Potential roles of key microbial players in the strawberry greenhouse with soil amendments

**Fig 5:**
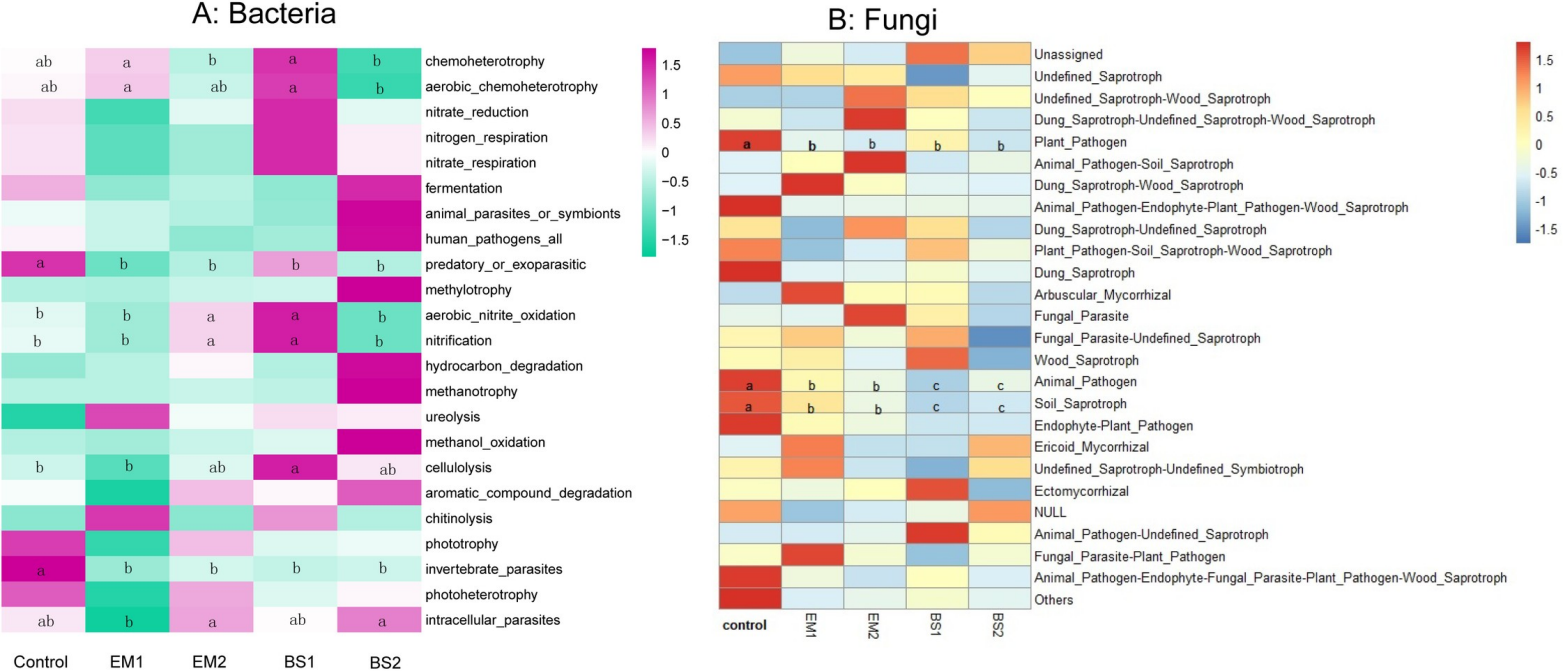
Functional prediction analysis. Heatmap showing relative functional abundance predictions of the bacterial communities based on FAPROTAX (A), and fungal communities based on FUNGuild (B). The color code represents the row z-score. Different letters (a, b, ab, c) represent statistical significance at P <0.05.

The FAPROTAX database has been extensively used to analyse the biogeochemical cycling processes of bacterial communities based on confirmed characteristics of isolated and cultured bacteria. We assigned 768 out of 3,863 bacterial OTUs (19.9%) to at least one microbial functional group (S2 File). Sixty-seven predicted functions were identified. Then the most abundant 23 functional groups were further evaluated for their relative profiles in the different soil samples (Fig 5A). Chemoheterotrophy and aerobic_chemoheterotrophy were the two highest relative abundance in different treatments among the putative functions, accounting for 15.55% and 14.90% of the total, respectively. Many functional groups in the soil were more abundant after BS1 and BS2 treatment and were reduced after EM1 and EM2 treatment. Among them, BS2 application significantly lowered the relative abundance of chemoheterotrophy, aerobic_chemoheterotrophy function. Whereas EM1, EM2 application remarkably reduced nitrogen_respiration and nitrate_respiration function. After the four soil amendments applications, there was a significant decline in the function of predatory_or_exoparasitic, invertebrate_parasites.

We performed FUNGuild to predicte the function of the fungal microbial community. A total of 825 OTUs were classified into fungal guilds, accounting for 66.86% of all OTUs. As shown in Fig 5B, Unassigned and Undefined_Saprotroph functional groups of fungi dominate the top 25 functional guilds, with average abundances of 54.27% and 40.45%, while other functions are less predominant (about 6%). All Soil amendments significantly reduced Plant_Pathogen, Animal_Pathogen and Soil_Saprotroph functional fungi; interestingly, for the inhibition of the latter two functions, the BS-based Soil amendments were more effective than the EM ones.

### 3.5. Structure and composition of bacterial co-occurrence networks

**Fig 6:**
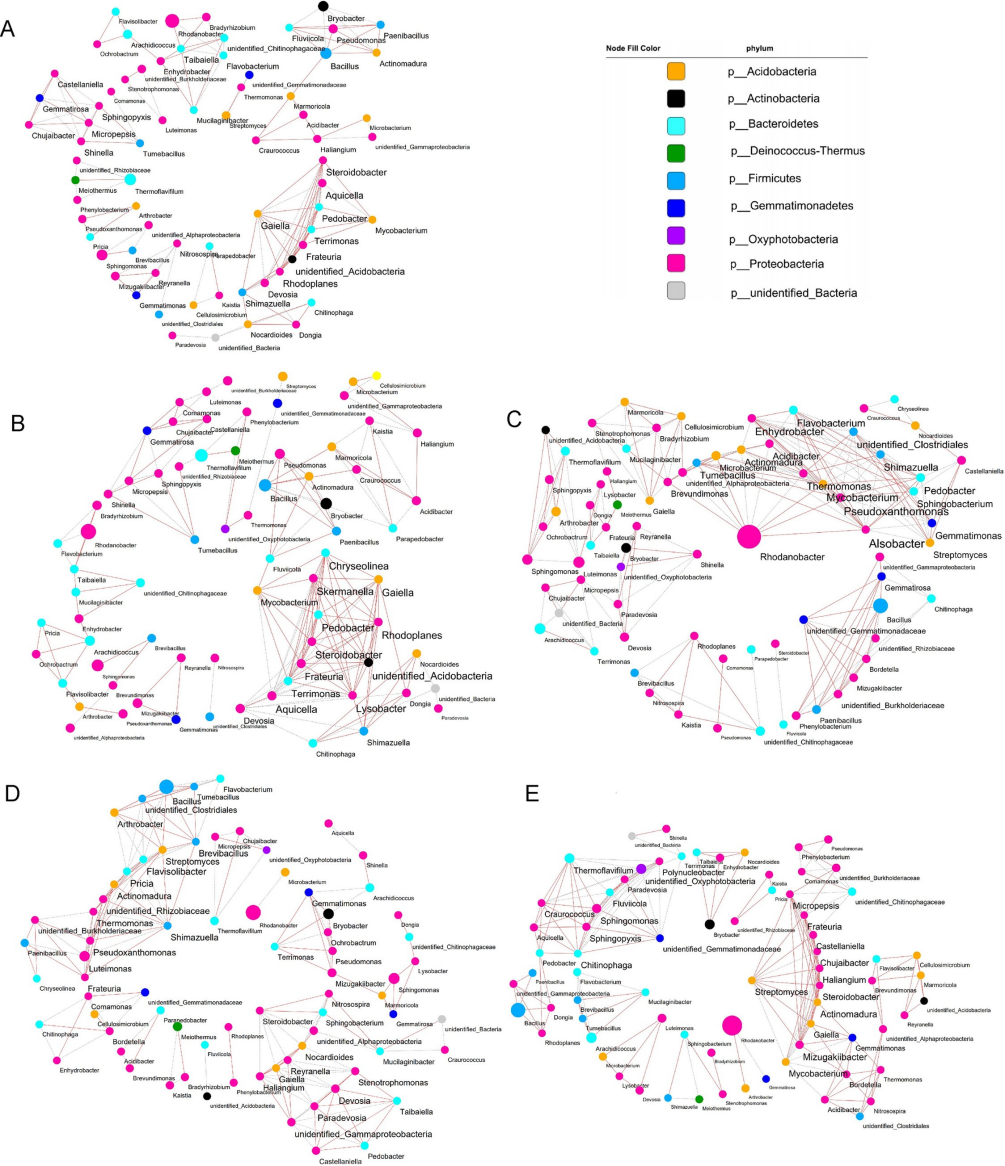
Co-occurrence network diagram of soil bacterial communities at genus level between different treatments. Based on Spearman correlation, Cytoscape was used to construct bacterial co-occurrence network. Correlation is shown as edge (positive correlation = gray; Negative correlation = light red), correlation coefficient r>|0.8|, and P <0.05. The size of nodes is positively correlated with relative abundance of genus, and the color of nodes is distinguished by phylum level.

Understanding the interactions between different microbial taxa in communities and their responses to environmental change is a central goal of ecology. Therefore, network analysis is widely used to greatly assist us in assessing soil ecology or their contribution to habitat niches. Here, co-occurrence network analysis was conducted to assess the complexity of the interactions among bacterial genera detected in strawberry soils treated with different amendments. Spearman was used to calculating the correlation between the top 80 bacterial genera in the soil. Then, we selected the Cytoscape software to visualize the co-occurrence network (Fig 6) and evaluate several vital topological properties (Table 1).

**Table 1:** Topological properties of correlation network.

Parameters	Control	EM1	EM2	BS1	BS2
Nodes	70	74	74	74	73
Total links	139	200	216	184	191
Positive links	84	131	107	95	133
Negative links	55	69	109	89	58
Clustering coefficient	0.798	0.584	0.700	0.683	0.731
Network density	0.058	0.064	0.080	0.068	0.073
Shortest paths	580 (12%)	1604 (29%)	1976 (36%)	918 (16%)	1268 (24%)
Network diameter	6	13	15	9	10
Average neighbors	3.971	4.676	5.838	4.973	5.233

Soil bacterial communities at genus level in different treatments.

In our model, each node represents a microbial genus. The links imply interrelationships between microbes (classified as positive or negative); in addition, the value of network density is often used to assess the robustness closeness of the overall microecology, which is positively correlated with others. The bacterial network was composed of nodes and edges, and there were 70–74 nodes with significant correlations. The results showed that nodes, total links, positive links, negative links, network density, network diameter and average neighbors all increased after the addition of soil amendments. We easily found that the bacterial network of EM2 was the most complex and compact, and it held the highest topological properties of total links (216) and network density (0.080); however, the simplest network (control) was only 139 and 0.058, respectively. The correlation characteristics of the soil bacterial network were more balanced (almost equal number of positive and negative links) in the EM2 treatment compared to EM1. Compared with BS1, positive links of soil bacteria in BS2 increased greatly, while negative links decreased.

Utilizing the connectivity degree between microbes, we sought to find the keystone genera within each network (S3 File). The degree of connectivity is based on the sum of the links between the genus and other taxa; thus, the higher the connectivity degree value, the closer the relationship between the genus and other taxa. By identifying species with high connectivity degree, we are more likely to explore key taxa in the community. Counting the number of bacterial genera with a connectedness degree greater than 8 in each treatment, it was surprising to find that they were 5, 11, 16, 12, 19 in Control, EM1, EM2, BS1, BS2, respectively. Specifically, the connectivity degree of *Gaiella* (p__Acidobacteria), *Rhodoplanes* (p__Proteobacteria) and *Steroidobacter* (p__Proteobacteria) in control soil were the highest, but they were all lower than those in other soil amendments. In EM1 soil, the degree values of *Lysobacter* (p__Proteobacteria), *Aquicella* (p__Proteobacteria) and *Rhodoplanes* (p__Proteobacteria) were the highest. In EM2 soil, the degree values of *Alsobacter* (p__Proteobacteria), *Pseudoxanthomonas* (p__Proteobacteria) and *Enhydrobacter* (p__Proteobacteria), *Flavobacterium* (p__Bacteroidetes), *Rhodanobacter* (p__Proteobacteria) were the greatest, and it was noted that the relative abundance of *Rhodanobacter* was also the richest in the community. In BS1 soil, the degree values of, *Patricia* (p__Bacteroidetes) and *Flavobacterium* (p__Bacteroidetes), *Shimazuella* (p__Firmicutes) were the highest. In BS2 soil, the genera with the highest degree were *Mizugakiibacter* (p__Proteobacteria) and *Actinomadura* (p__Actinobacteria), *Chitinophaga* (p__Bacteroidetes).

## 4. Discussion

In this study, by using amendments synthesized by BCAs and compost, we have revealed details of the soil bacterial and fungal community structure through high-throughput sequencing. The diversity of microbial communities is an indicator of the effectiveness of agricultural practices [61]. Although the succession pattern of bacteria and fungi in monocropping strawberry fields remains unclear, soil degradation can be partly explained by changes in the diversity and structure of the microbial community. Some researchers found that the richness and diversity of bacterial and fungal communities would be greatly reduced with the increase of continuous cropping years (especially over five years) [50]. Adopting different soil amendments, EM2 and BS1 could promote bacterial diversity, while EM1 can significantly reduce soil bacterial richness. In addition, the alpha-diversity indices of soil fungal communities did not change significantly among treatments. Previous studies have shown that EM application improved soil microbial diversity [62, 63]. This is similar to the results of the EM2 treatment in our study. It may be since EM bacterial agent is suitable for a higher proportion of compost, and the number of unique OTUs of bacteria with higher compost increased significantly in the same BCAs (Fig 2A). Recent studies have shown that the application of BCAs of *Bacillus*-based formulates does not decrease the total microbial diversity and community [64]. Instead, *Bacillus subtilis* increased the bacterial diversity in tobacco rhizosphere soil [65], which is also verified by our findings.

The structures of microbial community have undergone profound changes. Proteobacteria were the most abundant bacteria associated with disease inhibition in the soil with long-term monostrophic fertilization [6]. In our study, most of the dominant bacteria with significant differences (P < 0.05) belong to Proteobacteria whose abundance increased to different degrees after adding soil amendments. This may be one of the factors that guarantee strawberry soil health. We illustrated that when soil amendments were introduced, the total relative abundance of dominant bacterial genera increases significantly from 15.83% (control) to 52.2% (EM1). However, among the genera of soil fungi (S3 Table), the total relative abundance of seven pathogens decreased with the application of soil amendments, with the greatest decrease in EM1, BS2. The relative abundance of *Fusarium*, the most well-known of the soil and plant pathogens, decreased significantly (p < 0.05) after adding soil amendments. Previous studies have shown that the application of BCAs and certain organic matter can effectively inhibit soil pathogens, including *Verticillium* sp, *Fusarium oxysporum* and *Penicillium digitatum* [66, 67]. Besides, it has been shown that *Bacillus* and *Trichoderma* (components of EM) can protect host plants against pathogens [68, 69]. In this study, the relative abundance of the corresponding microbial population (especially *Bacillus*) was significantly increased by adopting BS-based soil amendments. This suggests that soil amendments may lead to increased competition for resources and antagonistic between bacteria and pathogens in composting soils. Therefore, through positive interaction, the indigenous microbial population benefits from introducing microbes into soil systems.

The microbial community consists of a large number of abundant and rare taxa. In most ecosystems, the abundance of microbes contributes to microbial biomass and mineralization of organic matter [70]. In agricultural soils, microbial communities are affected by multiple factors such as sampling time, carbon and nitrogen sources, soil water content and plant physiological status [71]. These factors may be related to microbial community assembly. Both the dominant bacterial and fungal taxa were explained by environmental factor correlation analysis, indicating their high correlation with key soil physicochemical properties. Consistent with other results, our results showed that EM2, BS1 treatments occupied richer bacterial community, and there is significant correlation between the bacterial community and TN, AP, AK and C/N ratios, whereas the fungal community was only significantly correlated with AP and TK. Thus, the application of four soil amendment s reconstructed soil microbial communities through changes in soil physicochemical properties.

Studies have shown that green manure of soybean promoted the increase of functional bacteria like nitrogen-fixing bacteria, nitrifying bacteria and denitrifying bacteria in soil, indicating that green fertilizer application promoted the nitrogen fixation and nitrogen cycle process in soil [56, 72]. Based on FAPROTAX function prediction, we estimated that the BS-based soil amendments promoted multiple functions of soil bacteria, such as aerobic nitrite oxidation, nitrification and cellulolysis. Nevertheless, the EM-based soil amendments significantly reduced multiple functions of soil bacteria. Accordingly, FUNGuild prediction showed that soil amendments significantly reduced the taxa of Plant_Pathogen, Animal_Pathogen and Soil_Saprotroph functional fungi, especially the decrease of plant_pathogenic functions matched the decrease of these fungal pathogenic taxa shown in S3 Table. Consistently, the BS-based soil amendments developed showed more prominent inhibition of these deleterious pathogens than the EM-based soil amendments. Further analysis of the co-occurrence network of 80 dominant genera in soil microbial community showed that the interactions among soil bacteria after applying soil amendments were more complicated (increased topological properties) than that in the control soil. Additionally, the bacterial networks of the EM2-treated soils were the most balanced and complex. Based on these results, we hypothesize that the application of EM and more compost in strawberry soils promote connections within the bacterial community, which makes it more likely that specific bacteria establish symbioses in agricultural soils [59]. According to this hypothesis, the colonization rate of relatively single flora in BS-treated soil was lower than that of mixed flora EM, which maintained the health and balance of soil microbes weakly. In the bacterial network of different treatments, the keystone genera have undergone significant changes, but they all generally belong to Proteobacteria and Bacteroidetes. We observed that positive interactions between nodes indicated niche overlap, while negative interactions indicated competition or variation [35]. In this study, phylogenetically related microorganisms form well-differentiated clusters (Fig 6), and clusters with close correlations among key genera were mainly composed of positive correlation. These results are similar to the co-occurrence network of natural and agricultural soils [59]. In the bacterial network of EM-treated soil, the number of keystone genera and clusters were generally greater than other treatments. Despite a substantial increase in the relative abundance of *Bacillus* in BS treatment, it did not become a keystone genus in the microbial network, which further confirmed the previous hypothesis. However, whether these clusters constructed around key genera represent different functional groups remains obscure.

Nevertheless, the ecological effects of these soil amendments on strawberry cultivation need to be evaluated comprehensively, including determining the growth, yield and quality of strawberries under different treatments and even their long-term effects [11]. Meanwhile, our study was limited to a relatively small range of sites and could not consider deeper insights under larger-scale conditions with different physicochemical properties and climate types [8, 73]. Our studies have revealed the effects of soil amendments on bacterial community structure and co-occurrence networks in strawberry soil. However, the molecular mechanism, phenotypic characteristics, and interactions behind these changes and their effects on plant health remain unclear. Therefore, the q-PCR technique should be used to study how the absolute number of target microorganisms react to soil amendments in agricultural soils. Further metagenomic studies are needed to accurately determine the beneficial bacteria and pathogens at the species level.

## 5. Conclusion

In summary, our research showed that EM2/BS1-treated soil amendments significantly increased bacterial diversity, whereas they had no significant effect on fungal diversity. The effect of the four soil amendments on soil microbiome structure was significant, as all of them reduced the relative abundance of fungal pathogens including *Rhizopus*, *Penicillium* and *Fusarium*. FUNGuild predicted that soil amendments significantly reduced some detrimental functions of soil microhabitat systems (Plant_Pathogen, Animal_Pathogen). Besides, correlation analysis suggested that soil amendments could have an indirect effect on the soil microbial community by changing environmental factors. Moreover, all soil amendments enhanced the connectivity of bacterial networks, which was the most complex and balanced in EM2-treated soils. Therefore, EM2 and BS1, as novel soil amendments, have the potential to regulate soil microbial communities and promote sustainable agricultural development.

## Supporting information

S1 Fig Strawberry greenhouse and the appearance of Biological Control Agents (BCAs). The specific status and appearance of the two commercial BCAs (agent of EM and BS), and the scene of agricultural management greenhouse for strawberry plant.
(TIF)

S1 Table The physical and chemical properties of soils of different soil samples (means and standard deviations, n = 3). (DOCX)

S2 Table Analysis of the alpha diversity. Diversity indices of soil microbial communities based on 16S rRNA and ITS genes analyzed from sequencing.
(DOCX)

S3 Table Relative abundance percentage of dominant (>1% relative abundance) bacterial genera in strawberry greenhouse soils. (DOCX)

S4 Table Relative abundance percentage of pathogenic fungus detected in strawberry greenhouse soils. (DOCX)

S5 Table Mantel test showing the correlations between soil physicochemical properties and the bacterial and fungal abundant OTUs in soils. (DOCX)

S1 File OTU list for amplicon sequencing of soil fungi and bacteria. (XLSX)

S2 File Complete annotated data for FAPROTAX and FUNGuild function predictions. (XLSX)

S3 File Analysis of the keystone genera. Parameters of nodes (genera) in a bacterial co-occurrence network, including the connectivity degree between key microbes.
(XLSX)
