# Supplementary figures and images for: Response of soil microbiome structure and its network profiles to four soil amendments in monocropping strawberry greenhouse
Source: PLoS One. 2021 Sep 29;16(9):e0245180. doi: 10.1371/journal.pone.0245180 (PMC8480769; doi:10.1371/journal.pone.0245180)

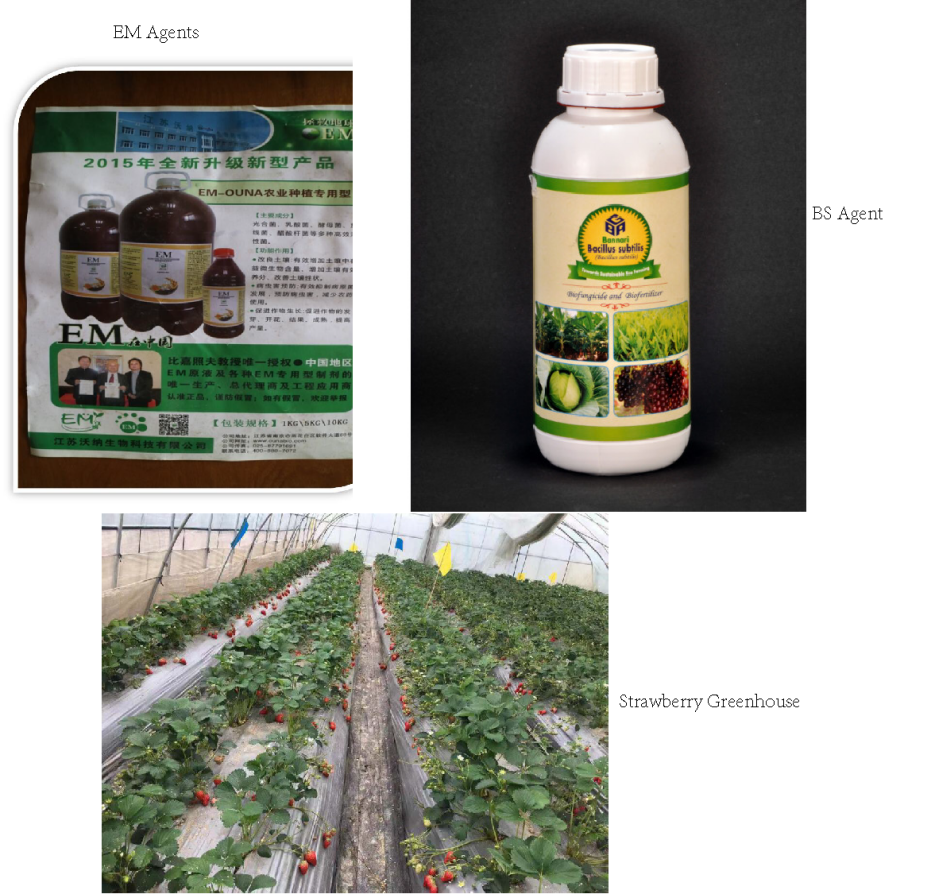

Supplement: S1 Fig — The specific status and appearance of the two commercial BCAs (agent of EM and BS), and the scene of agricultural management greenhouse for strawberry plant. (TIF) [file pone.0245180.s001.tif]
