# Supplementary material for: Response of soil microbiome structure and its network profiles to four soil amendments in monocropping strawberry greenhouse
Source: PLoS One. 2021 Sep 29;16(9):e0245180. doi: 10.1371/journal.pone.0245180 (PMC8480769; doi:10.1371/journal.pone.0245180)
Supplement: S1 Table — (DOCX) [file pone.0245180.s002.docx]

**S1 Table. The physical and chemical properties of soils of different soil samples(means and standard deviations, n=3 ) (DOCX).**

| treatments | pH | TOC  （g/kg） | TN  （g/kg） | C/N ratio | NH_4_^+^-N  (mg/kg) | NO_3_^-^-N  (mg/kg) | AP  (mg/kg) | TK  （g/kg） | AK  (mg/kg) |
| --- | --- | --- | --- | --- | --- | --- | --- | --- | --- |
| Control | 6.21±0.02^c^ | 38.07±1.141^b^ | 2.68±0.03^d^ | 14.21±0.20^ab^ | 3.84±0.12^d^ | 78.53±9.20^d^ | 29.61±1.23^d^ | 195.47±6.13^c^ | 13.35±0.16^b^ |
| EM1 | 5.92±0.02^e^ | 32.31±1.30^c^ | 3.58±0.15^b^ | 9.04±0.87^c^ | 11.81±0.45^c^ | 116.23±6.95^b^ | 44.26±0.40^a^ | 277.26±4.71^a^ | 16.03±0.51^a^ |
| EM2 | 6.06±0.04^d^ | 39.98±0.38^ab^ | 4.35±0.22^a^ | 9.20±0.15^c^ | 13.17±0.39^c^ | 142.46±4.13^c^ | 35.10±0.30^c^ | 246.16±3.08^b^ | 13.95±0.36^b^ |
| BS1 | 6.40±0.01^a^ | 40.51±0.64^ab^ | 2.69±0.12^d^ | 15.06±0.18^a^ | 15.41±0.86^b^ | 158.67±6.57^a^ | 34.24±0.58^c^ | 200.55±4.96^c^ | 15.43±0.19^a^ |
| BS2 | 6.31±0.02^b^ | 42.50±2.60^a^ | 3.05±0.15^c^ | 13.93±0.18^b^ | 21.68±0.50^a^ | 146.07±5.83^b^ | 37.60±1.71^b^ | 246.49±14.20^b^ | 13.88±0.46^b^ |
