# Supplementary material for: Response of soil microbiome structure and its network profiles to four soil amendments in monocropping strawberry greenhouse
Source: PLoS One. 2021 Sep 29;16(9):e0245180. doi: 10.1371/journal.pone.0245180 (PMC8480769; doi:10.1371/journal.pone.0245180)
Supplement: S2 Table — Diversity indices of soil microbial communities based on 16S rRNA and ITS genes analyzed from sequencing. (DOCX) [file pone.0245180.s003.docx]

**S2 Table. Analysis of the alpha diversity.** Diversity indices of soil microbial communities based on 16S rRNA and ITS genes analyzed from sequencing (DOCX).

| Classified | Treatments ID | Reads | OUT  Numbers | coverage | Diversity Index | | | |
| --- | --- | --- | --- | --- | --- | --- | --- | --- |
|  |  |  |  |  | Chao | ACE | Shannon | Simpson |
| Bacteria | Control | 72494 | 1554±42^b^ | 0.979 | 2125±264^ab^ | 2071±84^ab^ | 7.75±0.07^b^ | 0.981±0.004^a^ |
|  | EM1 | 85323 | 1294±36^c^ | 0.982 | 1767 ±40^c^ | 1808±46^c^ | 6.66±0.03^c^ | 0.951±0.011^b^ |
|  | EM2 | 86004 | 1877±77^a^ | 0.984 | 2328±171^a^ | 2435±101^a^ | 8.26±0.3^a^ | 0.989±0.004^a^ |
|  | BS1 | 85639 | 1857±57^a^ | 0.983 | 2388±36^a^ | 2404±40^a^ | 8.34±0.13^a^ | 0.991±0.001^a^ |
|  | BS2 | 87386 | 1647±196^b^ | 0.982 | 2053±325^ab^ | 2118±327^ab^ | 7.76±0.51^b^ | 0.982±0.008^a^ |
| Fungi | Control | 80179 | 440±214^a^ | 0.999 | 480±212^a^ | 483±216^a^ | 4.18±1.47^a^ | 0.829±0.108^a^ |
|  | EM1 | 80148 | 393±148^a^ | 0.999 | 425 ±144^a^ | 434±143^a^ | 4.03±1.32^a^ | 0.826±0.103^a^ |
|  | EM2 | 80141 | 482±152^a^ | 0.999 | 534±166^a^ | 542±166^a^ | 4.77±1.39^a^ | 0.873±0.137^a^ |
|  | BS1 | 80235 | 574±16^a^ | 0.999 | 627±10^a^ | 633±9^a^ | 5.27±0.40^a^ | 0.927±0.014^a^ |
|  | BS2 | 80187 | 395±100^a^ | 0.999 | 499±145^a^ | 432±101^a^ | 3.92±0.67^a^ | 0.824±0.058^a^ |
