# Supplementary material for: Response of soil microbiome structure and its network profiles to four soil amendments in monocropping strawberry greenhouse
Source: PLoS One. 2021 Sep 29;16(9):e0245180. doi: 10.1371/journal.pone.0245180 (PMC8480769; doi:10.1371/journal.pone.0245180)
Supplement: S3 Table — (DOCX) [file pone.0245180.s004.docx]

**S3 Table. Relative abundance percentage of dominant (>1% relative abundance) bacterial genera in strawberry greenhouse soils (DOCX).**

| bacterial Genus | CK | EM1 | EM2 | BS1 | BS2 |
| --- | --- | --- | --- | --- | --- |
| *Rhodanobacter* | 8.07^c^ | 21.28^a^ | 18.83^a^ | 9.47^c^ | 16.08^b^ |
| *Bacillus* | 4.55^b^ | 6.19^ab^ | 8.01^a^ | 8.65^a^ | 9.11^a^ |
| *Arachidicoccus* | 1.83^c^ | 6.36^a^ | 3.12^b^ | 1.36^c^ | 3.63^b^ |
| *unidentified_Oxyphotobacteria* | 0.01^a^ | 0.01^a^ | 0.02^a^ | 0.01^a^ | 3.34^a^ |
| *Thermoflavifilum* | 4.97^a^ | 1.25^c^ | 0.89^c^ | 1.01^c^ | 2.92^b^ |
| *Sphingomonas* | 4.02^a^ | 1.71^c^ | 2.47^b^ | 4.27^a^ | 2.23^b^ |
| *Luteimonas* | 0.27^a^ | 1.15^a^ | 3.52^a^ | 3.46^a^ | 1.59^a^ |
| *Pseudomonas* | 1.85^a^ | 3.16^a^ | 0.44^a^ | 0.56^a^ | 0.92^a^ |
| *Bryobacter* | 3.57^a^ | 2.08^a^ | 1.99^a^ | 3.85^a^ | 3.13^a^ |
| *Arthrobacter* | 0.07^a^ | 1.67^a^ | 1.59^a^ | 0.90^a^ | 0.81^a^ |
| *Pedobacter* | 0.05^a^ | 1.61^a^ | 0.72^a^ | 0.58^a^ | 0.31^a^ |
| *Mizugakiibacter* | 1.34^a^ | 0.16^a^ | 0.38^a^ | 1.59^a^ | 0.99^a^ |
| *Pseudoxanthomonas* | 0.80^a^ | 0.59^a^ | 0.41^a^ | 0.45^a^ | 1.24^a^ |
| *Chitinophaga* | 0.10^a^ | 0.98^a^ | 0.33^a^ | 0.18^a^ | 0.22^a^ |
| *unidentified_Chitinophagaceae* | 0.55^a^ | 1.78^a^ | 1.34^a^ | 0.63^a^ | 0.91^a^ |
| *Meiothermus* | 0.86^a^ | 0.54^a^ | 0.73^a^ | 2.02^a^ | 0.55^a^ |
| *Streptomyces* | 1.21^a^ | 0.17^a^ | 0.35^a^ | 0.38^a^ | 0.24^a^ |
| *Microbacterium* | 0.23^a^ | 1.30^a^ | 1.29^a^ | 1.10^a^ | 0.78^a^ |
| *unidentified_Burkholderiaceae* | 1.04^a^ | 0.24^a^ | 0.33^a^ | 0.22^a^ | 1.12^a^ |
| *Total* | 35.40 | 52.20 | 46.74 | 40.00 | 44.11 |
