# Supplementary material for: Response of soil microbiome structure and its network profiles to four soil amendments in monocropping strawberry greenhouse
Source: PLoS One. 2021 Sep 29;16(9):e0245180. doi: 10.1371/journal.pone.0245180 (PMC8480769; doi:10.1371/journal.pone.0245180)
Supplement: S4 Table — (DOCX) [file pone.0245180.s005.docx]

**S4 Table. Relative abundance percentage of pathogenic fungus detected in strawberry greenhouse soils (DOCX).**

| Plant pathogenic genus（Fungus） | Control | EM1 | EM2 | BS1 | BS2 |
| --- | --- | --- | --- | --- | --- |
| *Aspergillus* | 6.547^b^ | 0.339^b^ | 10.10^a^ | 1.501^b^ | 0.513^b^ |
| *Rhizopus* | 1.571^ab^ | 0.227^b^ | 0.436^a^ | 0.851^b^ | 0.143^b^ |
| *Penicillium* | 0.343^ab^ | 0.227^ab^ | 0.44^a^ | 0.259^ab^ | 0.118^b^ |
| *Fusarium* | 0.71^a^ | 0.023^c^ | 0.081c | 0.248^b^ | 0.03^c^ |
| *Gibberella* | 0.032^a^ | 0.01^a^ | 0.05^a^ | 0.599^a^ | 0.023^a^ |
| *Alternaria* | 0.546^a^ | 0.001^b^ | 0.007^b^ | 0.014^b^ | 0.005^b^ |
| *Mucor* | 0.013^d^ | 0.041^c^ | 0.14^a^ | 0.11^b^ | 0.053^c^ |
| *Botrytis* | 0.023^ab^ | 0.003^b^ | 0.009^ab^ | 0.011^ab^ | 0.033^a^ |
| *Curvularia* | 0.055^a^ | 0.005^a^ | 0.008^a^ | 0.005^a^ | 0.005^a^ |
