# Supplementary material for: Response of soil microbiome structure and its network profiles to four soil amendments in monocropping strawberry greenhouse
Source: PLoS One. 2021 Sep 29;16(9):e0245180. doi: 10.1371/journal.pone.0245180 (PMC8480769; doi:10.1371/journal.pone.0245180)
Supplement: S5 Table — (DOCX) [file pone.0245180.s006.docx]

**S5 Table. Mantel test showing the correlations between soil physicochemical properties and the bacterial and fungal abundant OTUs in soils (DOCX).**

| Physicochemical properties | Bacteria | | Fungi | |
| --- | --- | --- | --- | --- |
|  | r | P | r | P |
| NO3--N | 0.343 | 0.081 | 0.053 | 0.38 |
| NH4+-N | 0.423 | 0.087 | 0.358 | 0.53 |
| pH | 0.208 | 0.262 | 0.146 | 0.480 |
| TOC | 0.014 | 0.908 | 0.245 | 0.908 |
| TN | 0.541 | 0.008 | 0.133 | 0.645 |
| AK | 0.203 | 0.003 | 0.178 | 0.253 |
| AP | 0.686 | 0.002 | 0.752 | 0.001 |
| TK | 0.758 | 0.061 | 0.621 | 0.002 |
| C/N | 0.493 | 0.037 | 0.212 | 0.079 |
